# Regenerative abilities of mesenchymal stem cells via acting as an ideal vehicle for subcellular component delivery in acute kidney injury

**DOI:** 10.1111/jcmm.15184

**Published:** 2020-04-13

**Authors:** Lingfei Zhao, Chenxia Hu, Fei Han, Junni Wang, Jianghua Chen

**Affiliations:** ^1^ Kidney Disease Center The First Affiliated Hospital College of Medicine Zhejiang University Hangzhou China; ^2^ Key Laboratory of Kidney Disease Prevention and Control Technology Hangzhou China; ^3^ National Key Clinical Department of Kidney Disease Hangzhou China; ^4^ Institute of Nephrology Zhejiang University Hangzhou China; ^5^ State Key Laboratory for Diagnosis and Treatment of Infectious Diseases The First Affiliated Hospital College of Medicine Zhejiang University Hangzhou China

**Keywords:** acute kidney injury, mesenchymal stem cell‐based therapy, subcellular component delivery

## Abstract

Cell‐to‐cell communication and information exchange is one of the most important events in multiple physiological processes, including multicellular organism development, cellular function regulation, external stress response, homeostasis maintenance and tissue regeneration. New findings support the concept that subcellular component delivery may account for the beneficial effects of mesenchymal stem cell (MSC)‐based therapy‐mediated protection against acute kidney injury (AKI). Through the secretion of extracellular vesicles (EVs), formation of tunnelling nanotubes (TNTs) and development of cellular fusions, a broad range of subcellular components, including proteins, nucleic acids (mRNA and miRNA) or even organelles can be transferred from MSCs into injured renal cells, significantly promoting cell survival, favouring tissue repair and accelerating renal recovery. In this review, we outline an extensive and detailed description of the regenerative consequences of subcellular component delivery from MSCs into injured renal cells during AKI, by which the potential mechanism underlying MSC‐based therapies against AKI can be elucidated.

## 1 INTRODUCTION

Acute kidney injury (AKI) is a relatively common clinical syndrome, defined as an abrupt decline in glomerular filtration accompanied by complications, such as heart failure, dysregulation of electrolytes and even multi‐organ failure.^1^ AKI remains a worldwide public health issue due to its increasing incidence, significant mortality and lack of specific target‐orientated therapy.^2^ It has been estimated that this disorder may affect more than 20% of patients in hospital settings, whereas the total occurrence all over the world can reach up to 13 million people per year.^3^, ^4^ Patients that develop AKI also bear a high risk of poor prognosis and the development of chronic kidney disease (CKD).^5^, ^6^ Despite the substantial morbidity and mortality and regardless of the numerous underlying causes associated to this illness, beyond supportive measures and renal replacement therapy, little can be done to facilitate healing of the compromised kidney in humans. Therefore, exploring for a new therapeutic intervention to facilitate tissue repair during AKI is an urgent need.

Recent studies have documented that mesenchymal stem cells (MSCs) are promising candidates for kidney repair.^7^ MSCs are fibroblast‐like, multipotent progenitor cells that can be easily isolated from various adult tissues, including bone marrow, adipose tissue and the umbilical cord; characteristically, they are capable of differentiation, regeneration and immunomodulation.^8^ It has been confirmed in several different experimental AKI models that the administration of MSCs can significantly improve kidney histologic and functional recovery.^9^, ^10^, ^11^ It has also been shown that the apoptosis of renal tubular epithelial cells (RTECs) is common during AKI. Whereas the administration of MSCs displays a significant renoprotective effect by diminishing RTEC apoptosis.^12^ Meanwhile, Zhang et al found that MSCs could accelerate the proliferation of endothelial cells, promoting angiogenesis and preventing microvascular dropout.^11^ In terms of inflammatory cells, MSCs have the ability to reduce the infiltration of both neutrophils and macrophages,^13^ decrease the proliferative and cytotoxic activity of NK cells,^14^ suppress the maturation and differentiation of dendritic cells,^15^ regulate T and B cells,^16^, ^17^ and turn macrophages from a pro‐inflammatory phenotype M1 to an anti‐inflammatory phenotype M2.^18^ By interacting with various types of injured cells during AKI, MSCs showed that they have a role in minimizing injury, promoting regeneration, eliciting an immunological balance and, finally, contributing to the alleviation of renal injury. However, how these beneficial signals are transferred is still not well clarified.

**FIGURE 1:**
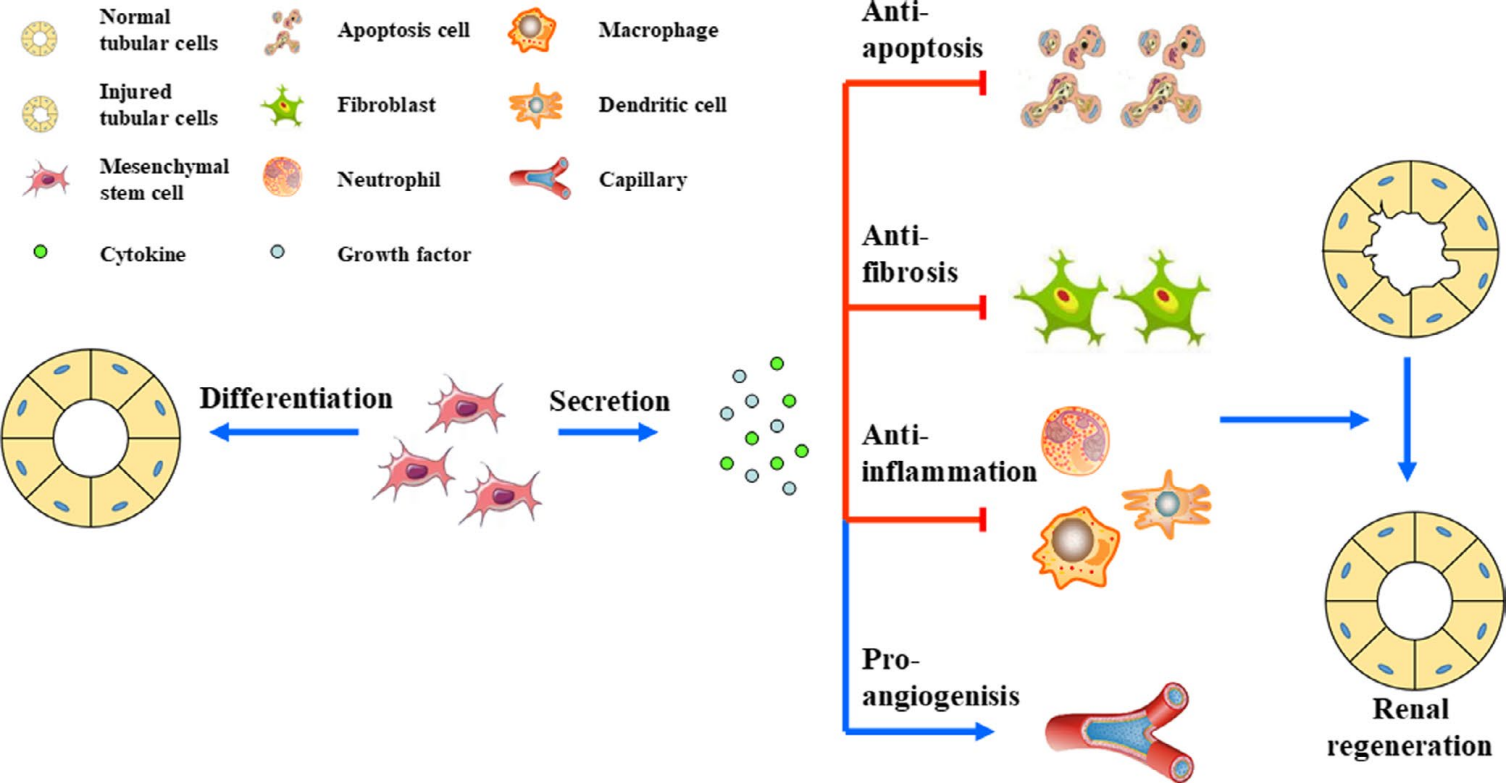
Mesenchymal stem cells' regenerative properties in AKI. It has been hypothesized that MSCs are able to directly differentiate into normal tubular cells or that they hold the capacity to secrete various kinds of cytokines and growth factors, presenting anti‐apoptotic, anti‐fibrotic, anti‐inflammatory and pro‐angiogenic effects, promoting renal regeneration. The latter is accepted by most investigators nowadays

Mainly, two hypotheses prevailed over the past decades related to the major repair mechanism underlying the protective effects of MSC‐based therapies for AKI (Figure 1). The first one suggested that injected MSCs migrated into the injured kidney, where they proliferated, engrafted and differentiated into normal kidney cells, promoting renal recovery. The second one proposed that the paracrine/endocrine activity of MSCs was responsible for their regenerative effects. However, the realization that the transient presence of MSCs within the injured kidney could neither account for cell differentiation nor for cell replacement led to an acceptance of the paracrine/endocrine‐dependent mechanism by most investigators.^19^ Commonly, it was thought that MSCs had the capacity to secrete a series of growth factors/cytokines presenting anti‐apoptotic, anti‐inflammatory, anti‐oxidative, and pro‐angiogenic effects and modulating host cells.^20^ Recent studies have suggested that MSCs may act as an ideal vehicle for subcellular component delivery, offering a new hypothesis on the mechanism underlying cell survival and tissue regeneration under the AKI stressful microenvironment. Subcellular component delivery is a widespread phenomenon observed throughout multiple mammalian cell types. Besides the delivery of small molecules, like proteins, RNAs or ions, over 40 variations of intercellular organelle deliveries have been described.^21^, ^22^ Cells under threat from extraneous stress need to maintain their homeostasis and reduce cell injury. Therefore, the acquisition of substances from neighbouring cells seems to be an important adaptation to the variable external environment and a classical example of multicellular cooperation. Emerging evidence indicates that the stress caused by exposure to a cytotoxic microenvironment is a major determinant for subcellular component delivery. Furthermore, the efficiency of the subcellular component delivery doubled when recipient cells were under certain conditions.^23^ In some circumstances, cell injury might even become an entire prerequisite.^24^ Compared with the paracrine/endocrine capacity hypothesis, direct subcellular component delivery by MSCs is a faster and more economical strategy for a renal regenerative process, as the biosynthesis of many transferred subcellular components, especially organelles, usually takes a longer time than that that can be afforded by damaged cells in crisis. Supporting this point, Zhang et al^25^ demonstrated that MSCs were therapeutically effective in enhancing burn healing through the transference of Wnt4 signalling related proteins. Besides, an earlier study showed that extracellular vesicles (EVs) secreted by bone marrow‐derived MSCs (BM‐MSCs) significantly mitigated acute lung injury (ALI) in mice by delivering FGF7 mRNA.^26^ As for organelles, the donation of mitochondria from BM‐MSCs to cardiomyocytes also played a critical role in the restoration of their energetic state.^27^

These results shed new light on the understanding of the complex and still not well‐clarified mechanism underlying MSC‐based therapy. Will this phenomenon be exploited as a new therapeutic approach for MSCs treating AKI? In this review, we offer an integrated view of those cellular structures and molecular mechanisms mediating subcellular component delivery. Then, we list all those different subcellular components known to be transferred, such as proteins, RNAs and even organelles, and analyse the outcome of subcellular component delivery during MSC‐based AKI therapies. Finally, we discuss how subcellular component delivery may be exploited as a new avenue for the treatment of AKI and those matters that still need to be solved in future studies.

## 2 CELLULAR STRUCTURES MEDIATING SUBCELLULAR COMPONENT DELIVERY IN MSCs

**FIGURE 2:**
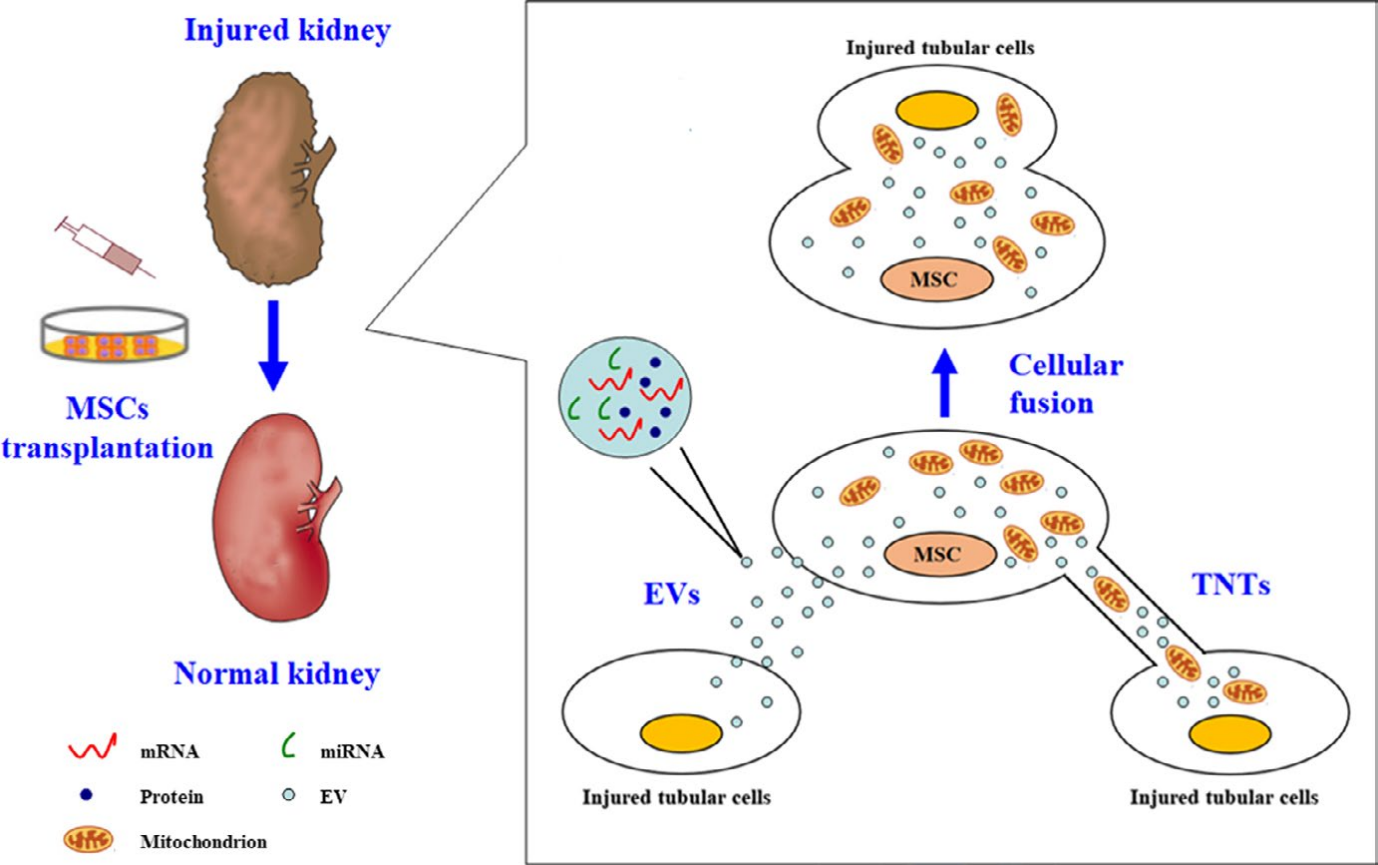
Cellular structures mediating subcellular component delivery in MSCs. MSCs are able to form a series of cellular structures to interact with target cells. EVs and TNTs as well as cellular fusion are the 3 major ones. Various subcellular components, like proteins, mRNAs, miRNAs and mitochondria, can be transferred by these mechanisms

Subcellular component delivery is based on the construction of several kinds of cellular structures between cells. Classically considered intercellular communication processes include ion channels, synaptic vesicles, gap junctions and paracrine receptor‐ligand bindings. However, the substances which MSCs deliver in the process of cell regeneration are not only biochemical signals but also defined subcellular components, such as proteins, RNAs and even organelles. In a sense, subcellular component delivery may be considered as a special form of intercellular communication that relies on particular cellular structures. EVs, tunnelling nanotubes (TNTs) and cellular fusion are the three major means for subcellular component delivery (Figure 2).

### 2.1 Extracellular vesicles

Extracellular vesicles are a heterogeneous population of biologically active membrane‐encompassed vesicles that can be secreted by almost every cell type, including MSCs, to the extracellular medium.^28^ According to their origin, size and molecular composition, EVs may be classified in exosomes (EXs), microvesicles (MVs) and apoptotic bodies. Although the components and loading mechanisms are still a matter of debate, it is widely accepted that EVs can act as a shuttle of cargoes, which include lipids, proteins, enzymes, coding and non‐coding RNA molecules (eg mRNAs, miRNAs and lncRNAs), and even organelles.^29^ After being secreted, EVs can interact with recipient cells, be internalized, and act as donors to release their biologically active molecules inside target cells, making a change on their function.^30^ Therefore, EVs can be regarded as envoys for long‐distance subcellular component delivery between cells.

Exosomes originated from MSCs (MSC‐EXs) (40‐100 nm in diameter) are generated from the endosomal network. Due to their small size, it has widely been accepted that they are responsible for transporting small molecular cargoes, such as proteins or genetic components like RNAs.^31^, ^32^ MVs derived from MSCs (MSC‐MVs) (100‐1000 nm in diameter) are formed by outward budding of the plasma membrane in a calcium‐ and calpain‐dependent manner; they are the largest among the different EV types. Consequently, besides transporting lipids, proteins, mRNAs and miRNAs, they also undertake the task of organelle delivery. To date, mitochondrial transport is the only well documented and understood organelle transfer mechanism by MSC‐MVs.^24^ However, in other cell types, it has been demonstrated that MVs can also transfer ribosomes.^33^ So, it is plausible to assume that other cytosolic structures may also be transported by MSC‐MVs.

In terms of apoptotic bodies, which also constitute a heterogeneous population of EVs, it was found that the perforin‐dependent apoptotic process of injected MSCs was essential to initiate MSC‐induced immunosuppression in both animal and mankind graft‐versus‐host disease (GvHD). This fact indicated that engulfed apoptotic bodies also had the ability to deliver subcellular components and explained their therapeutic effects.^34^

### 2.2 Tunnelling nanotubes

In the last 10 years, a burst of attention has been paid on TNTs, a newly discovered form of long‐distance cell contact structure. Morphologically, TNTs are defined as actin‐based ultrafine intercellular structures with diameters ranging from 50 to 200 nm and lengths which can span over several cell diameters.^35^ Before the determination of their composition, these tubular structures were believed to be extensions of the cell plasma membrane that formed an open‐ended conduit between two communicating cells. Biologically, the formation of TNTs seems to facilitate the transmission of cytoplasmic contents, not only of biological molecules, but also of selected organelles. Electrical signals, calcium signalling molecules and proteins are known to be transferred through TNTs.^27^, ^36^, ^37^ The motion of multiple organelles through these structures has also been visually confirmed by real‐time fluorescence microscopy observation.^38^, ^39^

The formation of TNTs has also been widely observed in MSCs. The vast majority of studies have reported that these structures are mainly established in ex vivo MSC coculture systems.^40^ However, a recent study by Li et al confirmed that MSCs could also utilize TNTs to transport mitochondria in an in vivo microenvironment. The transmission of mitochondria through TNTs helped in rescuing lung epithelium cells suffering from cigarette smoke injury.^41^ These facts indicated that TNTs are important, commonly found structures involved in MSC‐induced intercellular communication.

### 2.3 Cellular fusion

Cell contact by cell fusion is another important mechanism for subcellular component delivery. The merging of plasma membranes from two independent cells with intact nuclei allows a free exchange of cytosolic compounds and organelles. Though cell fusion is a rare event in mammalian cells, it definitely occurs when cells are incubated with stem cells.^42^ The subcellular component delivery and cell modification caused by cell fusion have important implications in the field of MSC‐based regenerative medicine. For instance, a study by Acquistapace et al suggested that a partial cell fusion with MSCs could reprogram cardiomyocytes into hybrid‐like cells bearing progenitor markers and transferred mitochondria. These cells regained their proliferative and survival properties, promoting tissue repair.^43^ However, based on the fact that few transplanted MSCs could be able to arrive around injured renal cells, the usefulness of this mechanism accounting for MSC‐based renal repair treatments still needs to be explored.

## 3 SUBCELLULAR COMPONENT DELIVERY PLAYS A CRITICAL ROLE IN MSC‐BASED THERAPY‐MEDIATED PROTECTION AGAINST AKI

We have demonstrated that there are different cellular structures that mediate subcellular component delivery between MSCs and target cells under different circumstances. Recent studies also found that MSCs were able to utilize this mechanism to alleviate AKI. In the following section, we will discuss about the involvement of subcellular component delivery in MSC‐based therapy‐mediated protection against AKI.

### 3.1 Delivery of miRNA

miRNAs are small non‐coding RNAs that are able to bind to the 3′ UTR of their target mRNAs, regulating gene expression at a post‐transcriptional level.^44^ It has been reported that miRNAs are involved in a wide range of biological and pathological processes, including cell proliferation, apoptosis, tumour development and stress response.^44^, ^45^ By transferring miRNA‐based information from stem cells to target differentiated cells, consequently, changing their phenotype, Yuan et al^46^ demonstrated that miRNAs could be responsible for cell regeneration. Regarding AKI, nephrologists suggested that MSCs might also had the capacity to reprogram renal cells towards a regenerative phenotype and alleviate cell injury by delivering the corresponding miRNAs (Table 1). The first study that showed that transferring specific miRNAs favoured renal repair was conducted by Gatti et al in 2011^47^. By pretreating EVs originated from MSCs (MSC‐EVs) with RNase to degrade their RNA cargoes, they found that the protective effects were abrogated, indicating that RNA molecules had a critical role in the regenerative potential of MSCs. Moreover, Collino et al used Drosha knock‐down, a specific miRNA depletion technique, in MSCs and observed a global tendency for miRNA down‐regulation in their EVs, while no changes in quantity, surface molecule expression and internalization ability of EVs were obtained compared to wild‐type MSC‐EVs. A reduction in Drosha expression largely impacted the functional and morphologic healing properties of MSC‐EVs in a glycerol‐induced model of AKI. At the molecular level, using RNA sequencing and gene ontology analysis, the authors postulated that MSC‐shuttled miRNAs were associated with the reversion of multiple gene alterations in kidneys after injury.^48^ Soon, an important question was raised: ‘Among the multiple kinds of miRNAs, which one is responsible for the regenerative effect?’.^49^ In order to answer this question, Lindoso et al cocultured MSC‐EVs with HK‐2 cells, a human renal proximal tubular epithelial cell (PTEC) line, in an in vitro model of ischaemia/reperfusion‐induced AKI (I/R‐AKI). An enhanced internalization of labelled MSC‐EVs in HK‐2 cells was observed with better protection from cell death. Then, the miRNA content in MSC‐EVs and HK‐2 cells was measured. The presence of miR‐148b‐3p, miR‐410, miR‐495 and miR‐548c‐5p was verified within MSC‐EVs. At the same time, an increase in the expression of these miRNAs in recipient cells was also observed, whether or not in the presence of the transcription inhibitor, actinomycin D. At the gene level, changes in the concentrations of miRNAs in HK‐2 cells were associated with downstream cell‐protective genes, such as CASP3 and CASP7, SHC1, and SMAD4. These facts suggested that the biological effects of MSC‐based therapies for AKI depended on direct miRNA transfer and followed post‐transcriptional modulation by MSC‐EVs.^50^ However, the above‐mentioned evidence only came from an ex vivo study, whether there exists a similar mechanism in in vivo systems is still unknown.

**TABLE 1:** Delivery of subcellular components underlying MSC‐base therapy‐mediated protection against AKI

Substances delivered	Reference	Year	Sample	Model	Stem cells source	Cellular structures	Delivered subcellular components	Outcome
Delivery of miRNA	Lindoso^50^	2014	PTECs	I/R	MSCs	EVs	miR‐148b‐3p, miR‐410, miR‐495 and miR‐548c‐5p	↑miR‐148b‐3p, miR‐410, miR‐495 and miR‐548c‐5p; ↑Cell viability
	Gu^51^	2016	Rats	I/R	WJ‐MSCs	EVs	miR‐30	↑miR‐30; ↓DRP‐1; ↓Mitochondrial fission; ↓Apoptosis; ↑Renal function
	Zhu^52^	2019	Mice	I/R	BM‐MSCs	EXs	miR‐199a‐3p	↑miR‐199a‐3p; ↓Sema3A; ↑AKT and ERK signalling activation; ↓Apoptosis; ↑Renal function
Delivery of mRNA	Ragni^53^	2017	PTECs	Cisplatin	BM‐MSCs	EVs	IL‐10 mRNA	↑IL‐10; ↑Cell viability
	Tomasoni^54^	2013	PTECs	Cisplatin	BM‐MSCs	EXs	IGF‐1R mRNA	↑IGF‐1R; ↑Cell proliferation
	Ju^56^	2015	Rats	I/R	UC‐MSCs	MVs	Human HGF mRNA	↑HGF; ↑ERK1/2 signalling activation; ↓Apoptosis; ↑Proliferation; ↓Fibrosis; ↑Renal function
	Du^57^	2013	Rats	I/R	WJ‐MSCs	NM	Human HGF mRNA	↑HGF; ↓Tubular EMT; ↓Fibrosis;
	Bruno^59^	2009	Mice	Glycerol	BM‐MSCs	MVs	Human POLR2E mRNA	↑POLR2E; ↑Proliferation, ↓Apoptosis, ↑Renal function
	Bruno^60^	2012	Mice	Cisplatin	BM‐MSCs	MVs	Human POLR2E mRNA	↑POLR2E; ↓Apoptosis; ↑Renal function
	Choi^61^	2014	Mice	I/R	K‐MSCs	MVs	mRNA	↑Proliferation; ↑Anti‐apoptosis; ↑Angiogenesis; ↓ Microvascular rarefaction; ↑Renal function
Delivery of proteins	Wang^63^	2018	HK‐2 cells	Cisplatin	UC‐MSCs	EXs	14‐3‐3ζ	↑14‐3‐3ζ; ↓Apoptosis; ↑Proliferation; ↑Autophagy
	Yuan^64^	2017	Rats	I/R	iPSC‐MSCs	EVs	SP1	↑SP1‐SK1–S1P signalling pathway; ↓Necroptosis; ↓Oxidative stress; ↓Pathological score; ↑Renal function
	Hagiwara^67^	2008	Rats	I/R	BM‐MSCs	NM	TK	Expression of human TK; ↓Apoptosis; ↓Inflammatory cell infiltration; ↓ROS; ↓Tubular injury scores; ↑Renal function
Delivery of organelles	Liu^74^	2014	HUVECs	I/R	MSCs	TNTs	Mitochondion	↓Apoptosis; ↑Cell viability

Abbreviations: AKI, acute kidney injury; BM‐MSCs, bone marrow‐derived MSCs; EMT, epithelial‐mesenchymal transition; EVs, extracellular vesicles; EXs, exosomes; HGF, hepatocyte growth factor; HUVECs, human umbilical vein endothelial cells; I/R, ischaemia/reperfusion; IGF‐1R, insulin‐like growth factor‐1 receptor; iPSC‐MSCs, induced pluripotent stem cells derived MSCs; K‐MSCs, kidney‐derived MSCs; MSC, mesenchymal stem cell; MVs, microvesicles; NM, not mentioned; PTECs, proximal tubular epithelial cells; ROS, reactive oxygen species; SP1, specificity protein 1; TK, tissue kallikrein; TNTs, tunnelling nanotubes; UC‐MSCs, umbilical cord‐derived MSCs; WJ‐MSCs, wharton's jelly‐derived MSCs.

Gu et al found that I/R‐AKI caused a decreased expression of miR‐30 in rats' kidneys but MSC‐EVs treatment entirely reversed this reduction. After using a specific miR‐30 antagonist to eliminate miR‐30 in MSCs, the expression of miR‐30 was comparable between the MSCs group and the vehicle group, regardless of the detection of MSC‐EVs within tubules. These results indicated that the increased level of miR‐30 in MSC‐treated rats originated from exogenous MSC‐EVs. Next, to explore the target gene and the related biological effect of the transferred miRNA, they measured the expression of DRP1 and evaluated the mitochondrial function. As they expected, mitigation of DRP1 activation and mitochondrial fragmentation was seen in MSC‐EVs–treated group, in parallel with the anti‐apoptotic and renal protective effects. Finally, they identified a miR‐30 delivery‐related pathway during MSC‐based therapy on AKI.^51^ A recent article, published in 2019, also revealed that the delivery of miR‐199a‐3p from EXs secreted by BM‐MSC (BM‐MSC‐EXs) into renal cells could down‐regulate Sema3A and activate the AKT and ERK pathways, finally inhibiting cell apoptosis and preserving renal function.^52^

### 3.2 Delivery of mRNA

Besides the ‘miRNA sponge’ property of MSCs, another paradigm is thought to be that MSCs may shuttle functional mRNAs that can be horizontally transferred and translated into proteins in target cells, activating tissue regenerative programs (Table 1). Ragni et al labelled MSC‐EVs with the membrane‐dye PKH26 and cocultured them with human proximal tubular cells HKC8. After a 24‐hour incubation period, 100% of the labelled EVs were engulfed by HKC8 cells. Moreover, the expression of the IL‐10 protein was detected in HKC8 cells, from which it was originally absent; interestingly, IL‐10 mRNA, but not the protein, was initially present in MSC‐EVs. These results indicated that IL‐10 mRNA was successfully delivered from MSC‐EVs to HKC8 cells. Clearly, the acquisition of IL‐10 mRNA and its translation into IL‐10 protein within HKC8 cells played a role in rescuing cell viability after cisplatin injury.^53^ Similarly, Tomasoni et al found that the insulin‐like growth factor‐1 receptor (IGF‐1R) mRNA is selectively shuttled into BM‐MDCs EXs. Then, this mRNA can be transferred into cisplatin‐damaged PTECs, where it is translated into the IGF‐1R protein, stimulating cell proliferation.^54^ Based on these ex vivo outcome, some in vivo studies were performed.

The hepatocyte growth factor (HGF) is a growth factor that exerts paracrine effects to promote cell regeneration and injury repair in multiple organs and in AKI.^55^ Data obtained by Ju et al showed that in I/R‐AKI rats that received human MSC‐MVs therapy, human HGF mRNA could be found in damaged rat tubular cells, indicating mRNA transfer. Transferred human HGF mRNA was subsequently translated into the corresponding protein. High HGF levels activated the ERK1/2 signalling pathway, while inhibited cell apoptosis, decreased collagen deposition and thrived cell proliferation, accelerating renal recovery.^56^ Similarly, the beneficial effect of HGF mRNA transfer in AKI was observed in an AKI‐CKD transition model by the same group.^57^ Aberrant incomplete repair after AKI was thought to be a main contributor to CKD.^58^ In this study, down‐regulation of the tubular epithelial‐mesenchymal transition (EMT) and retarded fibrogenesis was demonstrated, supporting the hypothesis that direct delivery of mRNA was a novel regenerative mechanism for MSC‐based therapies against AKI.^57^

Glycerol‐AKI is another common animal AKI model where the mechanism of mRNA delivery by MSCs has also been observed. Human POLR2E mRNA and its translated protein were confirmed to be localized in tubules of mice with AKI treated with MSC‐MVs, but not in those from the control group. The accumulation of extraneous components was indeed beneficial for the restoration of the injured renal function.^59^ In 2012, a group of researchers confirmed that this phenomenon was also observed in a mice model of cisplatin‐AKI, which was also a main type of AKI.^60^ Above‐mentioned evidence is all related with renal tubular cells; but, besides tubular cells, abnormalities in endothelial cells are also involved in the pathophysiological process of AKI. Solid evidence has demonstrated that there is an effective mRNA transfer from kidney MSC‐MVs to target endothelial cells. Both in GFP‐MVs–treated human umbilical vein endothelial cells (HUVECs) and in the kidneys of GFP‐MVs–injected I/R‐AKI mice, it was possible to observe the expression of GFP‐labelled mRNA. Although the identity of these mRNAs was not explored, the pro‐angiogenic signals transferred by MSCs were confirmed to make a contribution for the alleviation of microvascular rarefaction and renal injury.^61^

### 3.3 Delivery of proteins

Transferred miRNAs or mRNAs need to be translated into proteins before presenting a biological function. But, is there a direct mechanism for the delivery of functional proteins underlying MSC‐based therapy (Table 1)?

The 14‐3‐3 protein family is implicated in the modulation of a variety of signalling pathways, such as PI3K and mTOR, thought to take an important part in regulating cell survival and autophagy.^62^ A study by Wang et al demonstrated that EXs from umbilical cord MSCs (UC‐MSC‐EXs) exerted a beneficial effect over HK‐2 cells suffering from cisplatin injury. This effect was due to the presence of 14‐3‐3ζ proteins in these EXs, according to gain‐ and loss‐of‐function tests. However, whether 14‐3‐3ζ proteins were transferred into HK‐2 cells or their paracrine/endocrine ability had a remote effect over injured cells is a question that should be deeply investigated. A replenishment of 14‐3‐3ζ from UC‐MSC‐EXs was observed in 14‐3‐3ζ knock‐down HK‐2 cells; it was accompanied by promoted cell proliferation and inhibition of cell apoptosis by the activation of autophagy. All these evidences suggested that 14‐3‐3ζ proteins transferred by UC‐MSC‐EXs were the main reason for the therapeutic effect of UC‐MSC‐EXs on cisplatin injured HK‐2 cells.^63^

Yuan et al also found that EVs secreted from induced pluripotent stem cell‐derived MSCs (iPSC‐MSCs) could ameliorate I/R‐AKI in vivo and that this effect was due to the inhibition of cell necroptosis. To take a deeper look into the mechanism underlying these protective effects, they conducted a series of experiments. First, they incubated HK‐2 cells under hypoxia/reoxygenation conditions. Necroptosis of HK‐2 cells during injury was largely reduced when cells were pre‐treated with EVs from iPSC‐MSC (iPSC‐MSC‐EVs); the fluorescent marker that belonged to EVs could be observed in HK‐2 cells, suggesting the endocytosis of EVs by HK‐2 cells. Second, to assess whether there existed any substance transfer from EVs to HK‐2 cells, they measured the specificity protein 1 (SP1) content in HK‐2 cells. SP1 levels were dose‐dependent and increased after iPSC‐MSC‐EVs treatment. However, SP1 mRNA levels were unchanged, suggesting the direct transfer of SP1 from EVs into HK‐2 cells. Then, based on chromatin immunoprecipitation and luciferase assays, they found that the expression of the *SK1* gene was transcriptionally up‐regulated after being stimulated by SP1. Moreover, the formation of sphinganine‐1‐phosphate (S1P), a protein derived from SK1 was also increased, indicating the SP1‐SK1‐S1P signalling pathway was critical for the anti‐necroptosis effect over HK‐2 cells in vitro. Altogether, these results proved that iPSC‐MSC‐EVs could directly deliver SP1 into target renal cells, activating the SP1‐SK1‐S1P signalling pathway, which inhibited cell necroptosis and alleviated renal dysfunction.^64^

Besides transferring intrinsic proteins, MSCs can also be utilized as a vehicle to introduce specific gene‐modified proteins into renal cells, regarded as a superior strategy in protection against AKI. Tissue kallikrein is a serine proteinase that can cleave low molecular weight kininogen to release the vasoactive kinin peptide.^65^ As both the B1 and B2 kinin receptors are fundamental in restricting cell apoptosis and ameliorating renal dysfunction during I/R‐AKI, Hagiwara et al investigated the potential of tissue kallikrein‐modified MSCs (TK‐MSCs) in I/R‐AKI rats.^66^ After systemic TK‐MSCs injection, expression of human tissue kallikrein was found in rat glomeruli, indicating a successful delivery. Functionally, less cell apoptosis, an inhibition of inflammatory cell infiltration, reduced reactive oxygen species (ROS) levels, decreased tubular injury scores and better renal function were all observed in the TK‐MSCs group as compared with the unmodified MSCs group. These results suggest that the delivery of tissue kallikrein by MSCs may provide high benefits in protection of renal injury.^67^

### 3.4 Delivery of organelles

Transferring defined intracellular organelles such as mitochondria is another special form of intercellular communication that pose MSCs as an ideal vehicle for subcellular component delivery in rescue of injured cells.^21^

Mitochondria participate in multiple biological processes, like ATP production, energy metabolism, calcium signalling and cellular apoptosis that regulate cellular homeostasis.^68^ Mitochondrial dysfunction is a common element in several pathophysiological statuses. Defects in mitochondria can induce abnormalities in oxidative phosphorylation, which is coordinated with a wide range of stress responses, such as autophagy or regulated necrosis, helping to remove damaged cells.^69^ Contrary to the old view that mitochondria were only originated from maternal inheritance, current studies raised the concept that mitochondria have the ability to traverse cell boundaries and thus be horizontally transferred between cells.^70^ The active transfer of healthy mitochondria from MSCs can restore aerobic metabolism and protect cells from elimination. The first evidence suggesting that MSCs acted as mitochondrial donors in rescuing cell fate was obtained by Spees et al in 2006. Cells with defective mitochondrial function by mtDNA depletion did not survive in standard conditions unless they were cocultured with MSCs from which they could acquire healthy mitochondria and experience a recovery of their mitochondrial function.^71^ In terms of AKI, mitochondria are fundamental organelles that play a central role in the development of the illness.^72^ Can mitochondria also be successfully horizontally transferred into kidney cells, reprogram cell metabolism and facilitate renal recovery?

Plotnikov et al^73^ confirmed that, under normal ex vivo culture media, transportation of mitochondria from MSCs towards renal tubular cells could be observed. Besides tubular cells, vascular endothelial cells are also injured during AKI. To assess whether incorporation of mitochondria was able to rescue the fate of vascular endothelial cells during AKI, Liu et al incubated HUVECs with MSCs in an in vitro I/R‐AKI system (Table 1). Compared with the control group, less cell apoptosis and increased cell viability were observed in the treatment group, together with a normalized balance between aerobic respiration and glycolysis, suggesting the re‐establishment of aerobic respiration. Then, to explore the mechanism underlying mitochondrial protective effects, they incubated HUVECs with MSCs having abnormal mitochondria or when the formation of TNT‐like structures was inhibited. The protective effects were largely suppressed in both of these two situations, indicating that an extraneously mitochondrial supplement may account for the therapeutic benefit. Finally, using laser scanning confocal microscopy, mitochondria were visibly found in the lumen of TNTs. All these evidence demonstrated that the therapeutic effects of MSCs in alleviation of I/R‐AKI were related to the formation of TNTs and intraluminal mitochondrial transport.^74^

## 4 CONCLUSION AND FUTURE PERSPECTIVES

As discussed in this review, the data mentioned above provide information about a newly discovered mechanism to explain how a limited number of migratory MSCs at the site of injury may contribute to an enhanced protective effect after AKI. Therefore, MSCs can act as an ideal vehicle for subcellular component delivery during the treatment of the illness. The transfer of proteins, mRNAs, miRNAs and even organelles from MSCs to injured renal cells aids in the maintenance of cellular homeostasis, accelerating the recovery from injuries. The ascertainment of this phenomenon not only helps to further elucidate the mechanisms underlying the benefits of MSC‐based therapies for AKI, but also opens a prospect for novel and feasible clinical applications of MSCs. Nevertheless, potential risk of immune rejection, adipogenic differentiation or tumorigenesis may still limit the clinical use of MSCs. However, the transplantation of isolated or artificial therapeutic components for treating AKI and other illnesses emerges as an off‐the‐shelf therapy and a lucrative future therapy strategy in regenerative medicine.

Despite the encouraging future of therapies based on subcellular component delivery for AKI, some unexplored challenges still need to be solved before achieving a routine application in clinical settings. First of all, there still exist doubts on whether subcellular component delivery is self‐sufficient for the repair or requires synergistic release of other paracrine effects, although one study indicated that these two processes work independently from each other.^75^ Furthermore, the optimum donor type, dose, packaging and development of delivery methods also remain as unanswered questions for medical intervention. Last but not least, the dangers of developing adverse effects should always be kept in mind.

In conclusion, current studies indicate that the phenomenon of subcellular component delivery is consistent with the beneficial effects of MSC‐based therapies seen on kidney recovery in animal models of AKI. We look forward to the application of MSC‐based therapies in the clinic, whereas a better understanding of the pathophysiology of AKI and a complete clarification of the mechanisms underlying the protective effects for MSC‐based therapies may further contribute significantly in improving the outcome in patients with AKI.

## CONFLICT OF INTEREST

The authors confirm that there are no conflicts of interest.

## AUTHOR'S CONTRIBUTION

LF Zhao and JH Chen contributed to the conception of the manuscript. LF Zhao and CX Hu were responsible for the literature review. LF Zhao, F Han and JN Wang drafted and revised the manuscript. All authors read and approved the final manuscript.
